# Effect of aridity on species assembly in gypsum drylands: a response mediated by the soil affinity of species

**DOI:** 10.1093/aobpla/plaa020

**Published:** 2020-05-25

**Authors:** Arantzazu L Luzuriaga, Pablo Ferrandis, Joel Flores, Adrián Escudero

**Affiliations:** 1 Department of Biology and Geology, Rey Juan Carlos University, Móstoles, Madrid, Spain; 2 Botanic Institute of UCLM, Botanic Garden of Castilla-La Mancha, Avda. de La Mancha, Albacete, Spain; 3 División de Ciencias Ambientales, Instituto Potosino de Investigación Científica y Tecnológica, San Luis Potosí, México

**Keywords:** Aridity gradient, assembly rules, community weighted mean (CWM), edaphic endemism, functional diversity, gypsum soil, Mediterranean, phylogenetic diversity, semiarid, soil affinity

## Abstract

Previous studies found that plant communities on infertile soils are relatively resistant to climatic variation due to stress tolerance adaptations. However, the species assemblies in gypsum soil habitats require further investigation. Thus, we considered the following questions. (1) Do harsher arid conditions determine the characteristics of the species that form plant assemblages? (2) Is the selection of the species that assemble in arid conditions mediated by their ability to grow on gypsum soils? (3) Is the selection of species that assemble in harsher conditions related to phylogenetically conserved functional traits? Perennial plant communities were analysed in 89 gypsum-soil sites along a 400 km climate gradient from the central to southeastern Iberian Peninsula. Each local assemblage was analysed in 30 × 30 m plots and described based on taxonomic, functional (soil plant affinity) and phylogenetic parameters. The mean maximum temperatures in the hottest month, mean annual precipitation and their interaction terms were used as surrogates for the aridity conditions in generalized linear models. In the hottest locations, the gypsophily range narrowed and the mean gypsophily increased at the community level, thereby suggesting the filtering of species and the dominance of soil specialists in the actual plant assemblies. Drier sites had higher taxonomic diversity. The species that formed the perennial communities were close in evolutionary terms at the two ends of the aridity gradient. The mean maximum temperatures in the hottest month had the main abiotic filtering effect on perennial plant communities, which was mediated by the ability of species to grow on gypsum soils, and thus gypsum specialists dominated the species assemblies in the hottest locations. In contrast, the perennial communities on gypsum soils were relatively resistant to changes in precipitation. Our findings suggest that the warmer environmental conditions predicted by global change models will favour gypsum specialists over generalists.

## Teaser

We found that the perennial communities on gypsum soils were relatively resistant to changes in precipitation; however, temperatures in the hottest month were the main factor responsible for the selection of the species that finally established to form communities. It should be noted that species adapted to grow on gypsum soils (i.e. gypsophites) dominated plant communities in the hottest locations. Our findings suggest that the warmer environmental conditions predicted by global change models will favour gypsum specialists over generalists.

## Introduction

Ecological assembly rules represent the biotic and abiotic processes that prevent particular species from occurring in realized assemblages (*sensu*Keddy 1992; Wilson 1999). They are the ultimate expression of the existence of niche differences and ecological filters that act hierarchically at different temporal and spatial scales (Gotzenberger *et al*. 2012; HilleRisLambers *et al.* 2012). Environmental filtering has been evaluated along fertility gradients (Spasojevic and Suding 2012; Gerhold *et al.* 2013; Price *et al*. 2014), but it has rarely been tested for particular restrictive soils such as serpentines (see Fernandez-Going *et al.* 2012) or gypsum soils (see Luzuriaga *et al*. 2012; 2015, and Peralta *et al*. 2019, for annual plants; Delalandre and Montesinos-Navarro 2018, for plant–plant interactions). This is surprising because a high proportion of the taxonomic plant diversity in many hotspots depends on these restrictive soil habitats (Médail and Quézel 1999; Cacho and Strauss 2014). Furthermore, the Intergovernmental Panel on Climate Change predicts that these special soil habitats will be greatly affected by intensified dryness (IPCC 2014), mostly because of their island-like structure (Luzuriaga *et al.* 2018). Gypsum outcrops are widespread restrictive soils throughout the world that mostly occur in arid and semiarid conditions (Herrero *et al.* 2009). Gypsum soils impose harsh abiotic conditions for plants (i.e. gypsophytes), and thus they provide an ideal model for analysing community assembly processes under special conditions (Escudero *et al.* 2015).

It is well known that edaphic specialists have stress-tolerant functional attributes (Grime *et al*. 2000; 2008; Escudero *et al.* 2015; Sianta and Kay 2019) and the assembly of plant species on gypsum soils depends greatly on the soil affinity or soil specialization characteristics of each species (Palacio *et al.* 2007; Luzuriaga *et al.* 2015 for gypsum soils; Fernandez-Going *et al.* 2012 for serpentine plant communities). The mechanism that induces the restrictive occurrence of some species on gypsum soils is called gypsophily (Merlo *et al.* 2009). Thus, we can assume that gypsophily integrates the characteristics that a species needs to become established in these harsh environments (for more details, see Escudero *et al.* 2015). The gypsum dependence of species appears to be connected with evapotranspiration (Escudero *et al.* 2015), so in the present study, we hypothesized that the degree of specialization by the species in an assembly should vary with climate. In order to test this hypothesis and evaluate whether the assembly rules shift along climate gradients, we used the gypsum preference of each species as a plant functional trait. To determine the soil preferences of each species, we used the species-specific gypsophily values assigned by a committee of expert botanists who quantified the soil preferences (Mota *et al*. 2011). This so-called gypsophily index (GI) represents a species-specific plant functional property that is crucial for species establishment (including survival, growth and reproduction) (Palacio *et al.* 2007), and its analysis using similar techniques to other plant functional traits may help us to understand community assemblies on restrictive substrates (see Luzuriaga *et al.* 2015). We combined this GI with tools that were originally developed for describing trait diversity patterns among communities in order to identify the filters (i.e. assembly rules) that operate on perennial plant assemblages along an aridity gradient in the Iberian Peninsula. Xerophily seems to be a major component for integrating the gypsophily syndrome (Escudero *et al.* 2015), so we expected that the gypsophily range (GR) of co-occurring species would narrow and that the mean community gypsophily values would increase at the aridest end of our climate gradient.

Phylogenetic diversity is a surrogate for the evolutionary relationships among the species that form an actual community and it provides valuable information about the contribution of trait evolution to the community structure and composition (Webb *et al.* 2002). Community phylogenetic analysis alone may only provide limited insights into the mechanisms responsible for forming plant assemblages (see Mayfield and Levine 2010; Weiher *et al.* 2011), but it may yield information regarding environmental filtering processes when combined with the interpretations of specific traits because phylogenetic diversity integrates the patterns found in the traits of all species (Kraft *et al.* 2007; Kembel 2009). It is assumed that closer species in evolutionary terms are usually more functionally similar compared with those that are more distantly related (Connolly *et al.* 2011; Gerhold *et al.* 2015; but see Violle *et al*. 2011). Consequently, according to the environmental filtering hypothesis (Keddy 1992), it is expected that the phylogenetic diversity will reduce if the environment becomes harsher because only species from certain clades may be adapted to these conditions.

The main aim of the present study was to determine how perennial plant communities on gypsum soils vary according to the climate conditions in terms of precipitation and the mean maximum temperatures in the hottest month. It is well established that both of these climate factors explain additional and complementary proportions of the variability in plant performance (Munson *et al*. 2011, 2013). Thus, we assessed the changes in taxonomic, functional and phylogenetic diversity along an aridity gradient in terms of the mean maximum temperatures and mean annual precipitation, which could also provide insights into the possible responses of these communities to ongoing climate change (see Butterfield 2015). The following questions were addressed in this study. (1) Do the harsher arid conditions (in terms of the mean maximum temperature in the hottest month and mean annual precipitation) in gypsum systems determine the characteristics of the species that form plant assemblages? (2) Is the selection of the species that assemble in more arid conditions mediated by their ability to grow on gypsum soils (i.e. soil affinity index)? (3) Is the selection of the species that assemble in harsher conditions related to phylogenetically conserved functional traits? Understanding the effects induced by climate on the plant community assemblies that inhabit restrictive soil types may be critical for predicting how this unique plant biota will respond to ongoing climate change.

## Methods

### Study area

We studied a climate gradient covering a geographical length of 400 km, which included all existing gypsum island regions from the centre to the southeastern tip of the Iberian Peninsula. In total, 89 sites were selected on soils derived from gypsum outcrops classified as Xeric Haplogypsid (Soil Survey Staff 1994), which were located on southerly oriented gentle slopes. In this study, the aridity gradient comprised the broadest possible range of arid conditions in the Iberian Peninsula compatible with gypsophilous vegetation (Rivas-Martinez and Costa 1970; Escudero *et al.* 2015). The mean annual precipitation ranged from 195 to 565 mm and the mean maximum temperatures in the hottest month ranged from 30.8 to 34.5 °C (www.aemet.com). Aridity was defined as the joint effect of less annual precipitation together with higher mean maximum temperatures in the hottest month, which is more critical for plant development than the mean annual temperatures in terms of water availability in the soil. The perennial vegetation comprised gypsophilous scrubland dominated by *Thymus lacaitae*, *Helianthemum squamatum*, *Lepidium subulatum* and *Gypsophila struthium* in the centre of Spain, and by *Teucrium libanitis*, *Herniaria fruticosa* and *Fumana hispidula* in the southeast [**see Supporting Information—Table S1****]**. The vegetation structure in our study system was typical of gypsum outcrops and drylands, where it comprised a matrix of plant patches on large areas of bare soil (Escudero *et al.* 2015), with total cover values ranging between 11 and 52.2 %.

### Sampling design

At each site, we established a square plot measuring 30 × 30 m within homogeneous vegetated areas. We assessed the composition and structure of perennial vascular plants using four 30-m long linear transects parallel to the slope and 8 m apart in each plot. For each transect, we recorded the intercept length of every perennial plant in contact with the transect line. Species-specific cover was estimated visually in 12 quadrats (1.5 × 1.5 m) placed in each plot, with three quadrats per transect line. The total plant cover in the whole plot was determined as the proportion of the four transects in contact with perennial shrubs. Total plant cover was included in the statistical models as an estimate of the productivity in each plot. Climate variables were estimated with the climate simulation model CLIMOEST (developed for the Iberian Peninsula) by inputting the altitude, geographic coordinates and hydrographic basin. This simulation model estimates climate parameters derived from monthly temperature and rainfall values over the latest 50 years (Sánchez-Palomares *et al*. 1999).

### Taxonomic, functional and phylogenetic diversity indices

Each taxonomic, functional, and phylogenetic diversity parameter was calculated at the plot level (30 × 30 m). Perennial diversity was calculated using the inverse Simpson index as follows:

$$\begin{array}{l} Inverse\;Simpson=\;\frac{1}{\overset{s}{\underset{i=1}{\sum }}p_{i}^{2}}, \end{array}$$

where *S* is the number of species at the plot level and *p*_*i*_ is the proportion of species *i*. The diversity index was computed using the *vegan* package (Oksanen *et al*. 2016) in R v.3.4.1 (R Development Core Team 2018).

In order to characterize the affinity of each species for gypsum soils, we used the GI for Iberian gypsophilous species proposed by Mota *et al*. (2011)**[****see Supporting Information—Table S1****]**. The GI ranges from 1 (species that avoid gypsum soils) to 5 (species strictly linked to gypsum substrates, which are also called gypsophytes). Species with GI values from 2 to 4 are called gypsovags, i.e. species that tolerate gypsum and that can be established effectively in these types of soils, but that usually occur on other substrate types as well. In our study, no species had a GI value of 1 because we actively selected gypsum soils for our sampling plots.

The affinity of species for gypsum soils was described at the community level using three parameters. In particular, we aimed to describe the range of soil affinity values in each species assembly (GR), the community mean gypsophily (CMG; weighted mean) and the gypsophily diversity (GD) in each actual assembly. These indices are not correlated with each other and they are independent of the species richness (Mason *et al.* 2005), and they allowed us to identify the main assembly processes related to the abundances of species (Mason *et al.* 2013). Mason *et al.* (2013) defined functional richness as the amount of a niche space filled by species in a realized assembly. Based on this definition, we computed the GR as analogous to the functional richness in order to quantify the amount of space used by each assemblage in the gypsophily gradient. We calculated the range of gypsophily values in each assembly as follows.

$$\begin{array}{l} GR=max\left(GI\right)-min\left(GI\right) \end{array}$$

The GR values can range between 0 (all species in the assemblage have the same GI) and 3 (at least two species in the assemblage have extreme GI values).

The CMG index is analogous to the community weighted mean index (Lavorel *et al.* 2008), where it weights the GIs of species based on their relative abundance in the assemblage to obtain a single community level value. CMG was calculated as follows:

$$\begin{array}{l} CMG=\overset{n}{\underset{i=1}{\sum }}\;(GI_{i}\times p_{i}), \end{array}$$

where p_i_ is the relative contribution of species *i* to the community and *GI*_*i*_ is the GI of species *i*. CMG values can range between 2 (only species with sporadic presence on gypsum soils in the community) and 5 (only gypsophytes in the community).

The GD was employed to describe the divergence of the plant species cover along the gypsophily gradient in each assemblage. It was computed as the Rao quadratic diversity (Botta-Dukát 2005):

$$\begin{array}{l} GD=\overset{S}{\underset{i,j}{\sum }}\;d_{ij}\times p_{i}\times p_{j}, \end{array}$$

where *S* is the species richness and *d*_*ij*_ is the difference in the GI values between species *i* and *j*, which was calculated as follows.

$$\begin{array}{l} d_{i,j}=\;{\left(GI_{i}-GI_{j}\right)}^{2} \end{array}$$

These three community-level functional parameters were computed using the FD package (Laliberté *et al.* 2014) in R (R Development Core Team 2018).

We calculated the standardized effect size for GR and GD in order to standardize these values and avoid bias related to the species number in each plot. The GR and GD values observed were compared with those obtained after 10 000 randomizations of the species abundances (see Mason *et al.* 2013, for more details).

A phylogenetic tree was built for all 111 identified species in our study using the *V.PhyloMaker* R package (Jin and Qian 2019). This statistical package attaches the species in a user-supplied list to a megatree of 74 533 vascular plant species where the branch lengths correspond to the evolutionary divergence time between branches. Three phylogenetic diversity indices were used: phylogenetic species variability (PSV), phylogenetic species richness (PSR) and net relatedness index (NRI). The PSV (Helmus *et al.* 2007) measures the phylogenetic relatedness of the species in a local assemblage, where the PSV equals 1 when all of the species in a sample are unrelated (i.e. a star phylogeny) and it approaches zero as species become more related in the phylogeny. PSR is the product of the PSV and species richness, and it can be considered as the species richness in a sample after discounting species relatedness. The PSR is maximized at the species richness of the sample and it decreases towards zero as the relatedness increases.

In general, the phylogenetic diversity of assemblages is correlated with the species richness, so we calculated the NRI values for our data. This index indicates whether the phylogenetic diversity in an assemblage is greater or less than expected regardless of the species richness (Webb 2000):

$$\begin{array}{l} NRI=-1\times \frac{MPD-MPDrnd}{sdMPDrnd}, \end{array}$$

where MPDrnd are the means of the mean pairwise distance (MPD) values based on 999 randomly generated assemblages and the sdMPDrnd are the standard deviations of the 999 MPDs obtained from those assemblages. Thus, negative NRI values indicate higher than expected phylogenetic diversity in an assemblage given the species richness of that assemblage. The random assemblages generated in the null models were generated with the independent swap algorithm by drawing the same number of species from the pool as the number of species in the observed community, where the observed community occupancy rates were fixed (Gotelli and Entsminger 2001). These phylogenetic diversity indices were computed at the plot level using the Picante package (Kembel *et al.* 2010) in R (R Development Core Team 2018).

### Statistical analysis

We constructed generalized linear models based on the taxonomic richness, inverse Simpson index, CMG, GR, GD, PSR, PSV and NRI indices. We used the mean maximum temperatures in the hottest month and the mean annual precipitation during the last 50 years, as well as the interaction terms between both variables, as surrogates for the aridity conditions in each plot. We checked for correlations between both explanatory variables in order to avoid any increase in the variance (Pearson’s correlation coefficient: *R*^2^ = 0.17, *t* = 1.6, and *P* = 0.11). Each model included the total plant cover in the plot as a surrogate of plant productivity in order to statistically control for the effects of productivity, thereby allowing us to evaluate the effects of temperature and precipitation regardless of the well-known productivity effects. The distribution error and link function with the best fits to our data were used for each generalized linear model (see Table 1).

**Table 1.** Chi-square values obtained by the generalized linear models for taxonomic, functional and phylogenetic diversity indices. Each model included the total plant cover in the plot as a covariable in order to statistically control for the differences in the vegetation productivity along the aridity gradient. T: Mean maximum temperatures in the hottest month; P: mean annual precipitation; GR: gypsophily range; CMG: community mean gypsophily index; GD: gypsophily diversity; PSR: phylogenetic species richness; PSV: phylogenetic species variability; NRI: net relatedness index. The error distributions (Family) and link functions assumed in the models are indicated. Id: identity link function; Log: logarithmic link function. The signs of the coefficients are shown in parentheses. *0.01 < *P* < 0.05; **0.001 < *P* < 0.01; ***P < 0.001.

	Family (link)	*T*	*P*	*T* × *P*	Cover
Taxonomic indices					
Richness	*Poisson (Log)*	1.06	2.6	0.9	(+) 10.9***
Diversity	*Gaussian (Id)*	(–) 9.3**	(–) 5.1*	2.3	0.97
Functional indices					
GR	*Gaussian(Id)*	(–) 6.3*	0.9	1.1	0.12
CMG	*Quasipoisson* *(Log)*	(+) 11.1***	0.2	0.1	0.2
GD	*Gaussian (Id)*	2.1	0.1	3.2	
Phylogenetic indices					
PSR	*Gaussian (Id)*	2.2	0.4	2.3	(+) 5.3*
PSV	*Gaussian (Id)*	1.9	0.7	(+) 4.5*	0.09
NRI	*Gaussian (Id)*	2.4	0.2	(–) 7.3**	0.7

## Results

**Figure 1.**
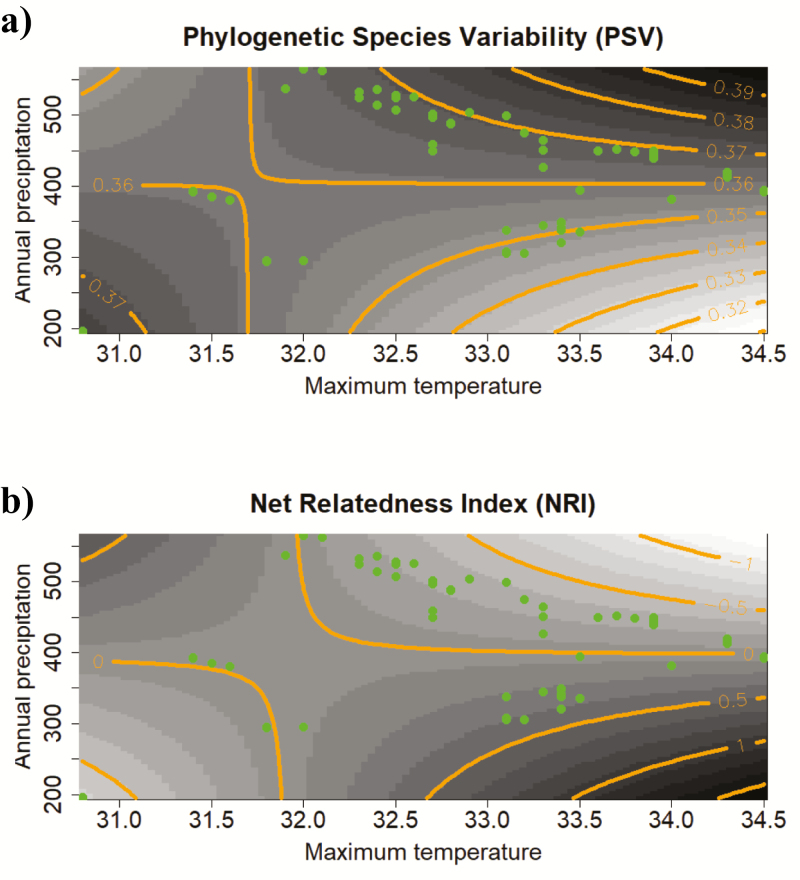
Graphic representation of the interaction between the mean maximum temperature in the hottest month and mean annual precipitation at the sampling locations based on: (A) the phylogenetic species variability (PSV) and (B) the Net Relatedness Index (NRI) in each plot. Circles represent the observed values. Lines join points with the same predicted PSV (A) or NRI (B) values based on our generalized linear models. Darker areas represent higher predicted values and lighter areas lower predicted values for PSV and NRI indices.

In total, 111 perennial plant species from 34 families were recorded along the aridity gradient on gypsum soils [**see ****Supporting Information—Table S1****]**. Sixteen species preferred gypsum soils and they appeared rarely on other substrate types, whereas the remainder were capable of growing on substrates other than gypsum. The mean maximum temperature and annual precipitation did not affect the taxonomic diversity and PSR after controlling for productivity effects by including the total vegetation cover in each plot in our models. However, we detected significant effects of the mean maximum temperatures on the inverse Simpson index, and on the functional and phylogenetic diversity indices (Table 1; Fig. 1). High mean maximum temperatures were related to lower taxonomic diversity and narrower ranges for the gypsophily indices. Thus, in the hottest environments, the GR narrowed at the community level. Furthermore, the CMG values showed that soil specialists (species with high gypsophily values) dominated the plant assemblages in hot conditions. In contrast, lower mean annual precipitation was associated with higher taxonomic diversity. Phylogenetic diversity was low at both ends of the gradient, at the most arid (high mean maximum temperatures and low precipitation) and at the least arid end (low mean maximum temperatures and high precipitation) (Fig. 1). Thus, the species that formed the actual perennial assemblages were closer in evolutionary terms at the most arid end of the gradient, and the species at the least arid end of the gradient where also phylogenetically closer to each other.

## Discussion

Precipitation is usually considered a primary driver of the vegetation dynamics in semiarid grasslands (Chesson *et al.* 2004; Miranda *et al.* 2011; Luzuriaga *et al*. 2012; 2015; Mulhouse *et al*. 2017; Peralta *et al*. 2019). However, we found that it did not affect the species richness and functional and phylogenetic diversity after statistically controlling for productivity effects. Our results agree with those reported by Fernandez-Going *et al.* (2012) who observed that the community composition on low-fertility serpentine soils generally varied less in response to precipitation than communities on more fertile soils. In addition, our findings support the prediction by Grime *et al*. (2000, 2008) that plant communities on nutrient-poor soils may be relatively resistant to changes in precipitation. This ability could be explained by the development of stress-tolerant functional traits (Damschen *et al.* 2012; Peralta *et al*. 2019), but other factors such as strong nutrient limitation may reduce the potential impact of changes in rainfall on plant growth. Other studies found that communities responded to a change in one resource only after the removal of the limitation due to another resource or condition (Klanderud and Totland 2005; Going *et al.* 2009). Nutrient availability and precipitation are closely linked because low precipitation is known to reduce the availability of nutrients due to water limitation restricting soil microbial processes (de Dato *et al.* 2006; Sardans *et al*. 2008) and decreasing the mobility of nutrients. Thus, plant adaptations to infertile soils are probably closely related to adaptations to low water availability conditions (Chapin 1991).

The taxonomic diversity decreased as the water availability increased, which may have been the combined result of two processes acting simultaneously: (i) many gypsophilous species do not fully exploit the water availability in rainier sites because their stress-tolerant nature will constrain their ability to utilize this resource and they may be displaced by calcophylous plant species (Dalgleish and Hartnett 2006; Bai *et al.* 2008; Nippert and Holdo 2015); and (ii) the higher water availability may benefit competitive species that are particularly efficient at exploiting resources and occupying space, such as the perennial tussock *Macrochloa tenacissima*. Thus, water limitation may be crucial for the maintenance of high plant diversity on gypsum soils in these systems, as shown by Eskelinen and Harrison (2015) who suggested that water and nutrient colimitation is the mechanism responsible for plant community resistance to changes in precipitation. Some studies have shown that communities on infertile soils are more diverse (Cacho and Strauss 2014), which may confer resistance to climatic variability by enhancing the potential for complementarity among the responses of species (Tilman and Downing 1994; McCann 2000).

Our results provide good evidence for the filtering effect of the mean maximum temperatures on gypsum plant communities (see also Moles *et al.* 2014, Butterfield and Munson 2016), where the relative abundance of gypsum specialist plants increased towards the hottest locations, regardless of the mean annual precipitation. Temperatures can induce moisture stress in many plant species (De Boeck *et al.* 2007) and gypsum specialists are equipped with stress-tolerant attributes (e.g. naked buds, deeply rooted tap roots, succulence and persistent soil seed banks; see Palacio *et al.* 2007; Escudero *et al.* 2015; Peralta *et al.* 2016), so heat may select the gypsophilous flora. Indeed, our results agree with those obtained in other studies of the distribution of strict gypsophiles (de Luis *et al.* 2019) and the regeneration stages of five gypsophiles (Sánchez *et al.* 2017). In general, gypsophily has been interpreted as an adaptive syndrome defined by traits that allow specialist plants to root in the hard physical crust (i.e. the physical hypothesis; Meyer 1986; Romao and Escudero 2005) and to cope with the ion imbalance that characterizes gypsisoils (i.e. the chemical hypothesis; Duvigneaud and Denaeyer-De Smet 1968; Boukhris and Lossaint 1975; Palacio *et al.* 2007). However, the participation of climate factors themselves in the modulation of gypsophily has received little attention, probably because it is broadly assumed that the gypsophilous flora is well adapted to stressful climate conditions. Future investigations should elucidate the effect of temperature on the modulation of gypsophily as an independent driver or as an environmental component that exacerbates other factors.

It should also be noted that the GD was not affected by the mean maximum temperature or annual precipitation, thereby suggesting that the actual local assemblages contained species that differed in terms of their tolerance of gypsum. Facilitation under the canopy of shrubs may have led to the presence of gypsovags beneath their canopies (Romao and Escudero 2005; Luzuriaga *et al.* 2015). Furthermore, the GD values were stable even thought there were remarkable shifts in the species composition along the aridity gradient (see Rivas-Martínez and Costa 1970), which was probably related to the divergent biogeographical history of the gypsum geological outcrops in the Iberian Peninsula (Herrero *et al.* 2009).

In this study, we found that evolutionarily close relatives coexisted in perennial plant communities (low phylogenetic diversity) at the most arid end of the aridity gradient (high mean maximum temperatures and low annual precipitation), and a similar situation occurred in the least arid locations (low mean maximum temperatures and high mean precipitation). These results were probably due to the intensification of different natural environmental filters operating at both extremes of the gradient. Thus, our results suggest that phylogenetic clustering might not have been induced by abiotic filtering under harsh conditions, but instead it could have been due to biotic interactions in more productive conditions. Bernard-Verdier *et al.* (2012) found similar trends in a Mediterranean rangeland. The coexistence of close relatives in resource-poor sites suggests that community membership is limited by a requirement for shared stress tolerance traits, i.e. habitat phylogenetic filtering (Grime *et al.* 2008; Butterfield *et al.* 2013). However, at the least arid end of the climate gradient, competition may have been a strong biotic filter, thereby leading to phylogenetic convergence via mechanisms for equalizing fitness (Chesson 2000; Grime 2006). Abiotic or biotic filtering may have relaxed as the aridity gradient changed to an intermediate level and this could have allowed the phylogenetic structure of the community to expand. According to the results obtained in the present study, the mean maximum temperatures had the main abiotic filtering effect on perennial plant communities, which was mediated by the ability of species to grow on gypsum soils (i.e. soil affinity index); thus, gypsum specialists dominated the species assemblies in the hottest locations. In contrast, the perennial communities on gypsum soils may have been relatively resistant to changes in precipitation. Therefore, our findings suggest that the warmer environmental conditions predicted by global change models may favour gypsum specialists (see de Luis *et al.* (2019) for an example of a strict gypsophile), thereby making the plant communities more gypsophilous in semiarid systems. However, several processes may interact, such as climate variability (Adler *et al.* 2006), extreme climatic events (Evans *et al.* 2011), nutrient availability (Delgado-Baquerizo *et al.* 2013), the soil microbial community (Zogg *et al.* 1997; Castro *et al.* 2010) and the trade-off between survival and plant growth (Lloret *et al.* 2012; Benavides *et al.* 2015), and thus the possible consequences for perennial plant communities are difficult to predict. In conclusion, the high taxonomic diversity of Mediterranean gypsum systems appears to be related to stressful arid conditions, and the tolerance of species for these conditions may be summarized using the GI for each species.

## Data Availability

Data used in this article are available at the URJC public repository under the following link: http://repositories.biodiversos.org/Luzuriaga_A.L/

## Supporting Information

The following supporting information is available in the online version of the article—

Table S1. Perennial plant species that occurred in the 89 plots located on gypsum soils and their gypsophily indices (GI).

plaa020_suppl_Suplementary_Table_S1
