# Supplementary material for: Effect of aridity on species assembly in gypsum drylands: a response mediated by the soil affinity of species
Source: AoB Plants. 2020 May 25;12(3):plaa020. doi: 10.1093/aobpla/plaa020 (PMC7288742; doi:10.1093/aobpla/plaa020)
Supplement: plaa020_suppl_Suplementary_Table_S1 [file plaa020_suppl_suplementary_table_s1.pdf]

Table S1. Perennial plant species that occurred in the 89 plots located on gypsum soils and their gypsophily indices (GI) (based on Mota *et al*. 2009 and Mota *et al*. 2011). 5: species strictly linked to gypsum substrates also called gypsophytes. 4: species that clearly prefer gypsum soils and rarely appear on other substrate types. 3: species that preferably grow on gypsum soils but that may also establish on other substrate types such as gypsiferous marls. 2: species appearing on gypsum soils that could grow on other substrate types without any problems; 1: species that avoid gypsum soils. We assigned a GI = 2 to species that did not appear in Mota *et al*. (2009) or Mota *et al*. (2011), since we only sampled gypsum soils we can assume that every species in our study was able to grow on gypsum substrates.

**Especies**	**Family**	**GI**
Ajuga chamaepitys	Lamiaceae	2
Allium sphaerocephalon	Liliaceae	2
Anthyllis cytisoides	Fabaceae	2
Arenaria cavanillesiana	Caryophyllaceae	3.33
Arisarum simorrhinum	Araceae	2
Artemisia herba-alba	Asteraceae	2
Asparagus albus	Liliaceae	2
Asparagus horridus	Liliaceae	2
Asphodelus aestivus	Liliaceae	2
Asphodelus ramosissimus	Liliaceae	2
Asphodelus albus	Liliaceae	2
Asphodelus fistulosus	Liliaceae	2
Astragalus granatensis	Fabaceae	2
Atractylis humilis	Asteraceae	2
Brachypodium retusum	Poaceae	2
Brassica repanda ssp gypsicola	Brassicaceae	4.4
Centaurea hyssopifolia	Asteraceae	5
Centaurium pulchellum	Gentianaceae	2
Centaurium quadrifolium	Gentianaceae	3.1
Cistus clusii	Cistaceae	2
Convolvulus althaeoides	Convolvulaceae	2
Coris hispanica	Primulaceae	4.75
Coris monspeliensis	Primulaceae	2
Cuscuta epithymum	Convolvulaceae	2
Cytinus hypocistis	Rafflesiaceae	2
Dipcadi serotinum	Liliaceae	2
Diplotaxis harra lagascana	Brassicaceae	2
Distichoselinum tenuifolium	Apiaceae	2
Dorycnium pentaphyllum	Fabaceae	2
Ephedra nebrodensis	Ephedraceae	2
Eryngium campestre	Apiaceae	2
Euphorbia serrata	*Euphorbiaceae*	2

Foeniculum vulgare	Apiaceae	2
Frankenia thymifolia	Frankeniaceae	3.36
Fumana ericoides	Cistaceae	2
Fumana procumbens	Cistaceae	2
Fumana hispidula	Cistaceae	2
Fumana laevipes	Cistaceae	2
Fumana thymifolia	Cistaceae	2
Gladiolus illyricus	Liliaceae	2
Globularia maritima	Globulariaceae	2
Gypsophila struthium ssp struthium	Caryophyllaceae	4.64
Helianthemum apeninum	Cistaceae	2
Helianthemum cinereum	Cistaceae	2
Helianthemum croceum	Cistaceae	2
Helianthemum hirtum	Cistaceae	2
Helianthemum squamatum	Cistaceae	4.83
Helianthemum alypodies	Cistaceae	5
Helianthemum syriacum	Cistaceae	2
Helichrysum stoechas	Asteraceae	2
Herniaria fruticosa	Caryophyllaceae	4.5
Inula viscosa	Asteraceae	2
Jurinea pinnata	Asteraceae	3.22
Koeleria vallesiana ssp castellana	Poaceae	3.86
Lapiedra martinezii	Amaryllidaceae	2
Launaea pumila	Asteraceae	3.64
Launaea fragilis	Asteraceae	3.1
Launaea nudicaulis	Asteraceae	2
Lepidium subulatum	Brassicaceae	5
Lithodora fruticosa	Boraginaceae	2
Lygeum spartum	Poaceae	2
Macrochloa tenacissima	Poaceae	2
Matthiola fruticulosa	Brassicaceae	2
Narcissus tortifolius	Amaryllidaceae	3.57
Odontites viscosa	Scrophulariaceae	2
Onobrychis stenorhiza	Fabaceae	2
Ononis tridentata ssp tridentata	Fabaceae	4.5
Ophrys speculum	Orchidaceae	2
Orobanche spp.	Orobanchaceae	2
Phagnalon saxatile	Asteraceae	2
Phillyrea angustifolia	Oleaceae	2
Phlomis crinita	Lamiaceae	2
Pinus halepensis	Pinaceae	2
Piptatherum miliaceum	Poaceae	2
Pistacia lentiscus	Anacardiaceae	2
Plantago albicans	Plantaginaceae	2

Polygala rupestris	Polygalaceae	2
Quercus rotundifolia	Fagaceae	2
Reseda stricta ssp stricta	Resedaceae	4.1
Reseda suffruticosa	Resedaceae	3.9
Reseda barrilieri	Resedaceae	3.14
Rhamnus lycioides	Rhamnaceae	2
Rhaponticum coniferae	Asteraceae	2
Rosmarinus officinalis	Lamiaceae	2
Salsola genistoides	Chenopodiaceae	2
Santolina chamaecyparisus	Asteraceae	2
Santolina viscosa	Asteraceae	3.67
Schoenus nigricans	Cyperaceae	2
Sedum gypsicola	Crassulaceae	3.44
Sedum sediforme	Crassulaceae	2
Sideritis fruticulosa	Lamiaceae	3
Sideritis leucantha	Lamiaceae	2
Stipa parviflora	Poaceae	2
Teucrium capitatum	Lamiaceae	2
Teucrium capitatum ssp gypsicola	Lamiaceae	4.5
Teucrium pumilum	Lamiaceae	4.63
Teucrium balthazaris	Lamiaceae	4.75
Teucrium gnaphalodes	Lamiaceae	2
Teucrium libanitis	Lamiaceae	5
Teucrium polium capitatum	Lamiaceae	2
Teucrium pseudochamaepitys	Lamiaceae	2
Teucrium turredanum	Lamiaceae	4.67
Thapsia villosa	Apiaceae	2
Thymelaea hirsuta	Thymelaeaceae	2
Thymus lacaitae	Lamiaceae	4.57
Thymus funkii funkii	Lamiaceae	2
Thymus hyemalis	Lamiaceae	2
Thymus moroderi	Lamiaceae	2
Thymus vulgaris	Lamiaceae	2
Thymus zygis	Lamiaceae	2
Urginea maritima	Liliaceae	2
